# An expression analysis of markers of radiation-induced skin fibrosis and angiogenesis in wound healing disorders of the head and neck

**DOI:** 10.1186/s13014-015-0508-3

**Published:** 2015-09-22

**Authors:** Steffen Koerdt, Nils H. Rohleder, Niklas Rommel, Christopher Nobis, Mechthild Stoeckelhuber, Steffi Pigorsch, Marciana-Nona Duma, Klaus-Dietrich Wolff, Marco R. Kesting

**Affiliations:** Department of Oral and Maxillofacial Surgery, Technische Universität München, Ismaninger Str. 22, 81675 Munich, Germany; Department of Radiation Oncology, Technische Universität München, Ismaninger Str.22, 81675 Munich, Germany

**Keywords:** Head and neck cancer, Radiation, Neck dissection, von –Willebrand factor, Procollagen-alpha, Tumor–growth factor-beta, Wound healing disorder

## Abstract

**Background:**

Radiation-induced fibrosis (RIF) is one of the severe long-term side effects of radiation therapy (RT) with a crucial impact on the development of postoperative wound healing disorders (WHD). The grades of fibrosis vary between mild to severe depending on individual radiosensitivity. In this study, we have investigated the molecular pathways that influence RIF and have correlated data from immunohistochemistry (IHC) for von –Willebrand Factor (vWF) and from Real-Time Polymerase Chain Reaction (RT-PCR) concerning markers such as Transforming Growth Factor (TGF)-**β**_1_, and vWF, with clinical data concerning the occurrence of WHD during follow-up.

**Methods:**

Expression profiles of the genes encoding TGF-**β**_1_, vWF, and α-procollagen (PC) were analyzed, by RT-PCR, in specimens from patients with (*n =* 20; 25.6 %) and without (*n =* 58; 74.4 %) a history of previous RT to the head and neck. Moreover, IHC against vWF was performed. Clinical data on the occurrence of cervical WHDs were analyzed and correlated.

**Results:**

A statistically significant increase in the expression profiles of α-PC and TGF-**β**_1_ was observed in previously irradiated skin samples (occurrence of RT >91 days preoperatively). vWF showed a statistically significant increase in non-irradiated tissue. Moreover, analysis of expression profiles in patients with and without WHDs during follow-up was performed. IHC showed a reduced amount of vessels and structural changes in epidermal tissue post-RT.

**Conclusions:**

The expression of markers of fibrosis and angiogenesis was analyzed in order to gain insight into molecular pathways that account for structural changes in irradiated skin and that eventually lead to WHDs. The results are congruent with reports from the literature and are a possible starting point for further research, as anti-TGF-**β**_1_ treatment, for example, could represent new therapeutic opportunities in the management of previously irradiated patients.

## Background

Whereas operative tumor resection is considered to be first line therapy in the primary treatment of limited head and neck malignancies (HNC), radiation therapy (RT) is one of the essential therapeutic supports. In particular, in an adjuvant scheme because of tumor size (T), the presence of lymph node metastasis (N), the local resection status (R), or the infiltration of lymph vessels (L), RT can significantly increase local tumor control and overall survival [1–4]. The risk of the development of lymph node metastasis during follow-up is also significantly reduced by means of RT [5–7]. Moreover, RT alone or in combination with chemotherapy is a fundamental option in cases of tumor recurrence or primarily inoperable tumors [8]. Doses of 66–70 Gray (Gy) are considered to be an effective in RT of the primary tumor and involved cervical lymph nodes [9].

However, radical tumor resection in combination with cervical lymphadenectomy, referred to as neck dissection (ND), is considered to be standard in surgical cancer treatment for cervical lymph node metastasis. Moreover, in situations with no clinical evidence for lymph node metastasis, histological studies have been able to show an occult occurrence of lymph node metastasis in up to 20–30 % depending on T and location, thereby establishing ND as an essential step in adequate oncological surgery of any invasive HNC [10–12]. In this method, the incision for cervical lymphadenectomy is placed 2 cm caudal from the mandibular margin, reaching from the sternocleidomastoid muscle region to the contralateral anterior belly of the digastric muscle. However, other surgical access to the neck, such as the McFee incision and the hockey stick incision, has been described, when a resection of the dorso-caudal levels is performed [13].

RT has profound effects, both acute and long-term, on skin and connective tissue. Wang and colleagues have been able to show the effect of RT on patients undergoing abdominal surgery in terms of a significantly reduced wound healing and increasing incidence of wound healing disorders (WHD) [14]. As preliminary studies by Rohleder et al. were able to show, blood velocity was significantly reduced in irradiated cervical skin at 2 mm depth [15]. However, blood flow parameters were increased in patients with the occurrence of a postoperative WHD. Even though these results seem to be surprising, a review of available literature revealed only very few studies, that investigated microvascular blood supply in the head an neck region, whereas tissues of other regions were subject to experimental analysis [16]. Our study group suggested two possible explanations: (i) On the one hand many vessels are being dissected during surgical interventions, leaving tissues that had an increased blood supply in the first place with reduced levels of perfusion postoperatively. The susceptibility to WHD increases respectively. (ii) On the other hand highly vascularized tissues have an increased risk for small postoperative bleedings, creating cervical hematomas and consequently enhance the risk for WHD [15].

The influence of RT on arteries and veins has been demonstrated to be dose-dependent [17, 18]. Schultze-Mosgau et al. have found no structural changes at doses of 40 to 50 Gy, whereas a dose of 60 to 70 Gy results in intimal dehiscence, hyalinosis, and a decreased ratio of the media to total vessel area in arteries [18, 19]. Moreover, RT also has an effect on connective tissue, as a compromised perfusion because of pericapillary fibrosis of the host site in microvascular tissue transfer, irregular capillary distribution, and a reduced amount of capillaries in the connective tissue have been demonstrated in animal models [19].

RT induces a reactive increased expression of cytokines such as Transforming growth factor (TGF)-**β**_1_. TGF-**β**_1_ plays an important role in wound healing by the stimulation or inhibition of fibroblast proliferation and microvessel formation [20–22]. Other studies have shown that the increased expression of TGF-**β**_1_ leads to significant fibrosis [23]. Increased expression levels of TGF-**β**_1_ in irradiated human and porcine skin have been detected in previous studies [24, 25]. However, other reports seem to be inconclusive, as *in-vitro* studies have demonstrated an inhibition of endothelial cell migration and proliferation, whereas a stimulation of microvessel formation has been shown *in-vivo* [26].

**Fig. 1:**
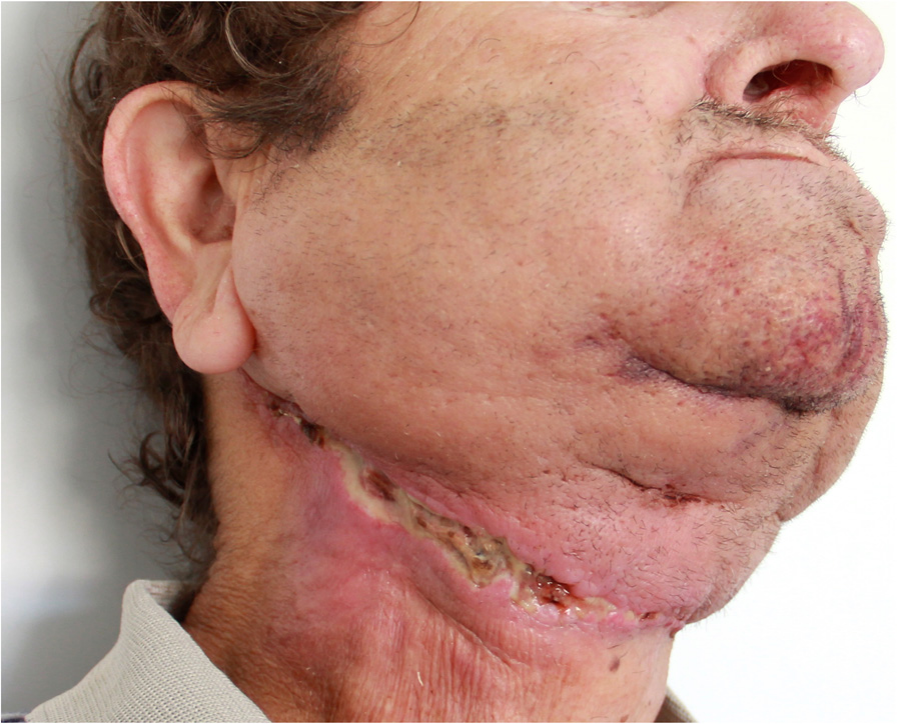
Cervical wound healing disorder (WHD) in a male patient (62 years) with a history preoperative radiation therapy with a total radiation volume of 64 Gy. The WHD is located in the field of the surgical approach to the neck dissection on the right side. A superficial suppurative dehiscence with a local inflammation on a length of approximately 10 cm can be observed. Dichotomized this is rated as the occurrence of a WHD in postoperative follow-up

This current study aims to identify the expression levels of TGF-**β**_1,_ von –Willebrand Factor (vWF) as an endothelial marker, and α-Procollagen (α-PC) as a marker of collagen formation and radiation-induced skin fibrosis in irradiated and non-irradiated cervical skin specimens, with special emphasis on postoperative WHDs as illustrated in Fig. 1. We have investigated expression levels by using histology, immunohistochemistry, and molecular biology in terms of real-time reverse transcription polymerase chain reaction (RT-PCR) and have correlated with the occurrence of WHD following ND. This study adds important information concerning the structural changes in irradiated cervical skin and the influence of the occurrence of WHD after surgery to the neck.

## Methods

### Patients and clinical data

We adhered to the Declaration of Helsinki on medical protocol and ethics, and the Institutional Review Board of the Technische Universität München, Germany approved the study (no. 3097/11). All participants were informed extensively and signed an informed consent agreement.

Inclusion criteria were defined as follows: (i) potential patients had to be inpatients at the Department of Oral and Maxillofacial Surgery, Technische Universität München, Germany between January 1, 2012 and December 31, 2012, (ii) availability of anamnestic data, (iii) patients had to undergo surgical procedures involving ND for HNC or other malignancies, and (iv) tissue specimens from the surgical access to the ND had to be available. A total of 78 patients were included in this study. All patients were treated by standardized surgical procedures in terms of ND, tumor resection, and reconstruction by using local or free flaps. Clinical data were obtained and documented before surgery (e.g., age, sex, history of RT, history of tobacco/alcohol abuse, etc.). Data on the occurrence of cervical WHD with respect to the location, size, onset after surgery, and the therapeutic interventions (frequent lavage, application of local agents, systemic antimicrobial treatment, operative debridement, etc.) were obtained during the first 30 postoperative days. All data was documented on a standardized from and any WHD were photographed.

During hospitalization, clinical data on the occurrence of cervical WHD (location, size, therapeutic intervention) was obtained at an interval of 30 days postoperatively. The length of stay in hospital and in the intensive care unit (ICU) was also documented. Radiation volumes were documented (range 50.4 to 69.9 Gy).

### Tissue samples

Skin samples were collected from incision margins during ND procedures under standardized conditions. Specimens with approximate dimensions of 2 × 10 mm were divided into two pieces under sterile circumstances, one of which was stored in Allproctect™-Solution (Qiagen, Germany) at −80 °C, whereas the other half was placed in 4.5 % buffered formalin for immunohistochemical staining.

### Histology/immunohistochemistry

Tissue specimens were embedded in paraffin, cut into 5-μm-thick sections and stained with Hematoxylin and Eosin (HE-stain) for histological orientation. According to local standard protocols, immunohistochemical staining for vWF was performed. The specimens were deparaffinized in xylene, rehydrated in a graded alcohol series followed by heat-induced epitope retrieval in Dako target retrieval solution (DakoCytomation, Denmark) for 20 min. After being washed with 0.05 M Tris-buffered saline (TBS), specimens were treated with 3 % H_2_O_2_ to block endogenous peroxidase and to avoid false positive results. Incubation of 5 % bovine serum antibody (PAA Laboratories, UK) was followed by treatment with primary antibody in a pretested concentration of 1:200 (DakoCytomation, Denmark; code No. A 0082). After incubation with the primary antibody, incubation with the secondary antibody (1:200, ant-rabbit; Vector Laboratories Inc., USA) for 60 min, and washes with TBS, streptavidin biotinylated horseradish peroxidase complex (Amersham Biosciences, Germany) was added to the sections. A diaminobenzidine (DAB) solution (Sigma-Aldrich, USA) was used for development, which was monitored and standardized for all stainings by using the specific antibody. Sections were counterstained with hematoxylin. Negative controls, in which the primary antibody was omitted, were treated alike otherwise. Immunohistochemical staining was analyzed by using a microscope, and images were captured with a digital camera (Nikon, Germany). The thickness of the epidermis was measured, and the amount of vessels was determined in 10 high power fields (HPF) within the specimen. All specimens were evaluated by two independent and specially trained investigators. At the time of analysis, the investigators were blinded.

### RT-PCR

Ribonucleic acid (RNA) isolation was performed by using the RNeasy® Protect Mini Kit (Qiagen). Beforehand, tissue samples were comminuted by using a rotor-stator system (Miccra, ART Labortechnik, Germany) and ultrasonification. After measurement of the amount of extracted RNA by means of a Biophotometer (Eppendorf, Germany), 1 μg isolated RNA was used for reverse transcription. Reverse transcription was performed according to the protocol of the SuperScript™ First Strand Synthesis System (Invitrogen, Germany). Random primers were used for RT. For RT-PCR, the cDNA sample, LightCycler® FastStart DNA Mater SYBR Green I reaction mix (Roche, Germany), forward and reverse primers, MgCl_2_, and RNase-free water, were analyzed by using the LightCycler® 1.0 system (Roche). Primer specifity was tested by using electrophoretic separation of the PCR product. Primer specifications are shown in Table 1. Amplification algorithms were as follows: 10 min at 95 °C, 40 cycles of 15 s at 94 °C, 10 s at 60 °C, and 10 s at 72 °C. A melting curve analysis was recorded in order to test for cDNA fragment consistency. The amount of RNA was automatically calculated by comparison of measured threshold cycles with standard curves and normalized with glyceraldehyde 3-phosphate dehydrogenase (GAPDH) as a housekeeping gene. A no template control was included in each run. All amplifications were carried out in triplicates.

**Table 1:** Primer sequences and GenBank® accession numbers^a^ of examined genes

Gene	Accession no.	Sequence 5´to 3		Efficiency in %^b^
Human procollagen alpha 1 (α-PC)	NC_000077.6	Forward	CTCGAGGTGGACACCACCCT	104.437
		Reverse	CAGCTGGATGGCCACATCGG	
Tumor growth factor beta 1 (TGF-β1)	NC_010448.3	Forward	TGGCGATACCTCAGCAACC	95.488
		Reverse	CTCGTGGATCCACTTCCAG	
Human von Willebrand Factor (vWF)	NM_000552	Forward	GTGACGTGTAATGGGAGACT	95.374
		Reverse	GTCATTGGCTCCGTTCTCAT	
GAPDH	NG_007073.2	Forward	GAGTCAACGGATTTGGTCGT	96.783
		Reverse	TTGATTTTGGAGGGATCTCG	

^a^GenBank® is the National Institutes of Health (NIH, USA) genetic sequence database, an annotated collection of all publicly available DNA sequences [38]

^b^χ=-1+10^(-1/slope)^

### Statistical analysis

All data were analyzed by using IMB®SPSS® for Mac (version 22.0; IMB Corp., USA). Means and standard deviation (SD) were calculated, and tests of significance were performed. For normally distributed values, t-test was performed. For values not normally distributed, the Mann–Whitney test was used. Statistical significance was defined as α = 0.05. All *p*-values are local and given as two-tailed.

## Results

### Clinical data

A total of 78 patients (33 males, 45 females) were included in this study. The mean age was 61.1 ± 11.7 years. A total of 20 patients received RT (25.6 %) before the surgical intervention because of tumor reoccurrence or previously non-surgically treated cancers in other centers. The majority of patients suffered from oral squamous cell carcinoma (*n =* 62; 79.5 %). Other less common diagnoses included malignant diseases of the salivary glands (*n =* 5; 6.4 %), sarcoma of the head and neck (*n =* 2; 2.6 %), CUP—syndrome and basalioma. Most patients (*n =* 65; 83.3 %) received immediate free flaps for reconstruction, such as radial forearm flaps (*n =* 30; 46.2 %), anterolateral thigh flaps (*n =* 14; 23.3 %), and osteocutaneous fibula free flaps (*n =* 10; 15.4 %).

No difference was evident between the groups with respect to age (*p* = 0.500), sex (*p* = 0.550), or alcohol (*p* = 0.422) and nicotine use (*p* = 0.521). The length of the in-hospital stay showed statistically significant differences between patients with and without a history of RT (24.1 ± 17.5 days vs. 13.4 ± 5.6 days; *p* = 0.000). However, the length of ICU treatment did not reveal differences between irradiated and non-irradiated patients (3.7 ± 6.3 vs. 1.78 ± 3.7; *p* = 0.103).

WHDs of the head and neck were also documented in clinical follow-up. Of 78 patients, 20 developed cervical WHDs (25.6 %). Generally, irradiated patients developed WHD earlier than those without a history of RT (5.9 ± 3.5 days post-op vs. 7.1 ± 3.3 days post-op; *p* = 0.429).

A multivariate analysis of clinical factors associated with the development of cervical WHDs was performed. The results are detailed in Table 2. Whereas neither age (*p* = 0.445) nor tobacco (*p* = 0.509) and alcohol (*p* = 0.346) use seemed to be statistically significant risk factors for the development of cervical WHG, an increased relative risk of 3.136 was associated with previous RT to the head and neck (*p* = 0.035). Other morbidities such as diabetes did not account for statistically significant differences.

**Table 2:** Multivariate analysis of results of factors associated with WHD

Factor	Patients, n (%)	WHD, n (%)	Odds ratio (95 % CI)	*p* Value
Age (yrs)				
≥70	21 (26.9)	7 (33.3)	1.536 (0.517–4.565)	.445
<70	57 (73.1)	14 (66.7)		
Tobacco use				
Yes	36 (46.2)	11 (52.4)	1.408 (0.516–3.841)	.509
No	42 (53.8)	10 (47.6)		
Alcohol use				
Yes	49 (62.8)	15 (71.4)	1.691 (0.572–5.003)	.346
No	29 (37.2)	6 (28.6)		
RT				
Yes	20 (25.6)	9 (45.0)	3.136 (1.059–9.292)	.035*
No	58 (74.4)	11 (55.0)		

Abbreviations: *WHD* wound healing disorder, *RT* radiation therapy, *CI* confidence interval

Data in parenthesis are percentages, unless noted otherwise. *statistically significant difference at α=0.05

Logistic regression analysis

### Histology/immunohistochemistry

**Fig. 2:**
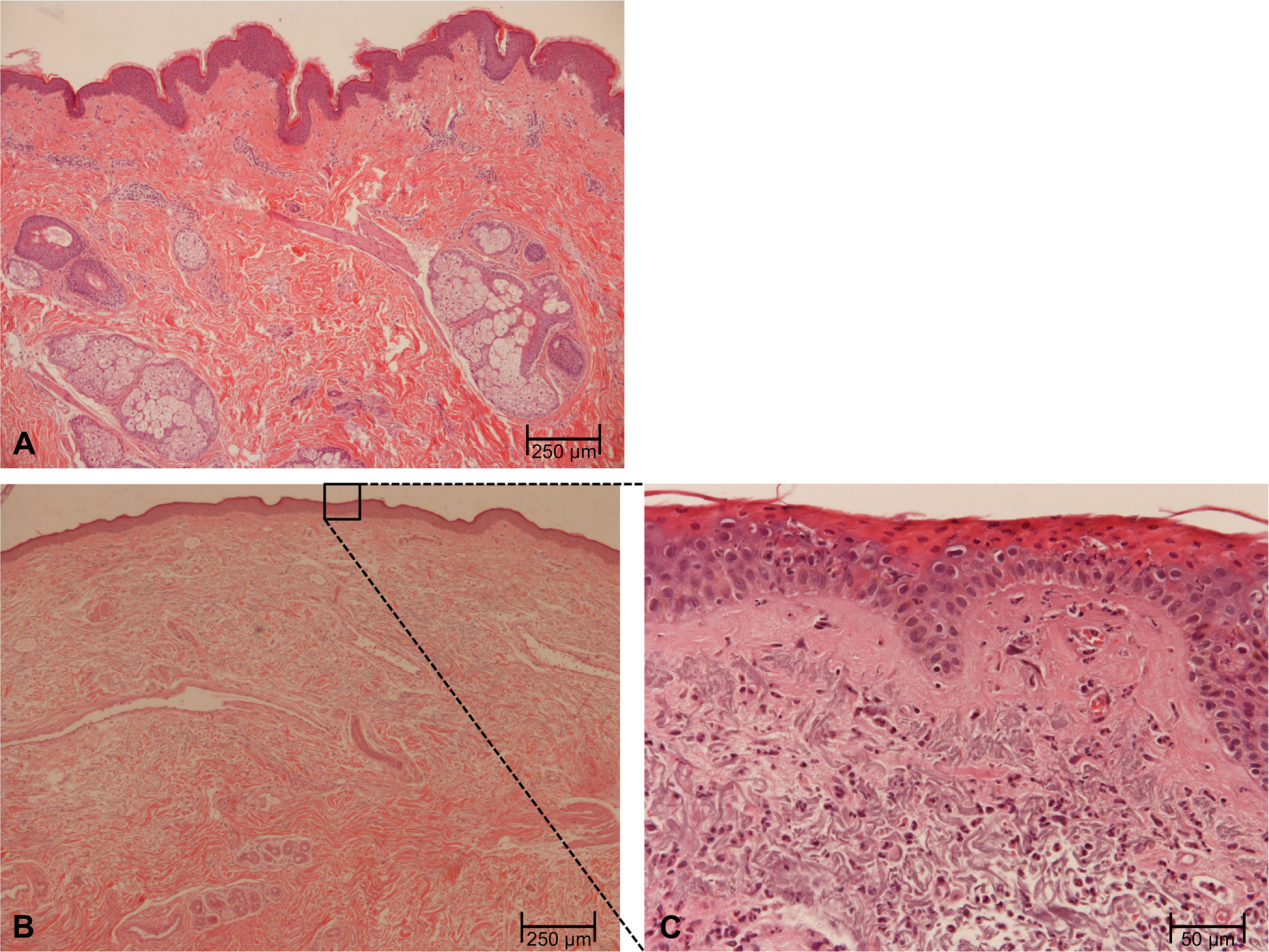
HE-Stain. In previously not irradiated tissue (**a**; scale bar = 250 μm) sebaceous glands, eccrine sweat glands, hair follicles as well as collagen and elastic fibres can be found in the epidermis**.** In irradiated tissue (**b**; scale bar = 250 μm) an epidermal atrophy with ratification of sweat and sebaceous glands could be found. No intact hair follicles could be identified. A detail enlargement (**c**; scale bar = 50 μm) shows hypereosinophilic superficial epidermal layers as well as inflammatory cells in the dermis. Not shown in this enlargement are hyperpigmentations in the epidermis and numerous melanophages

**Fig. 3:**
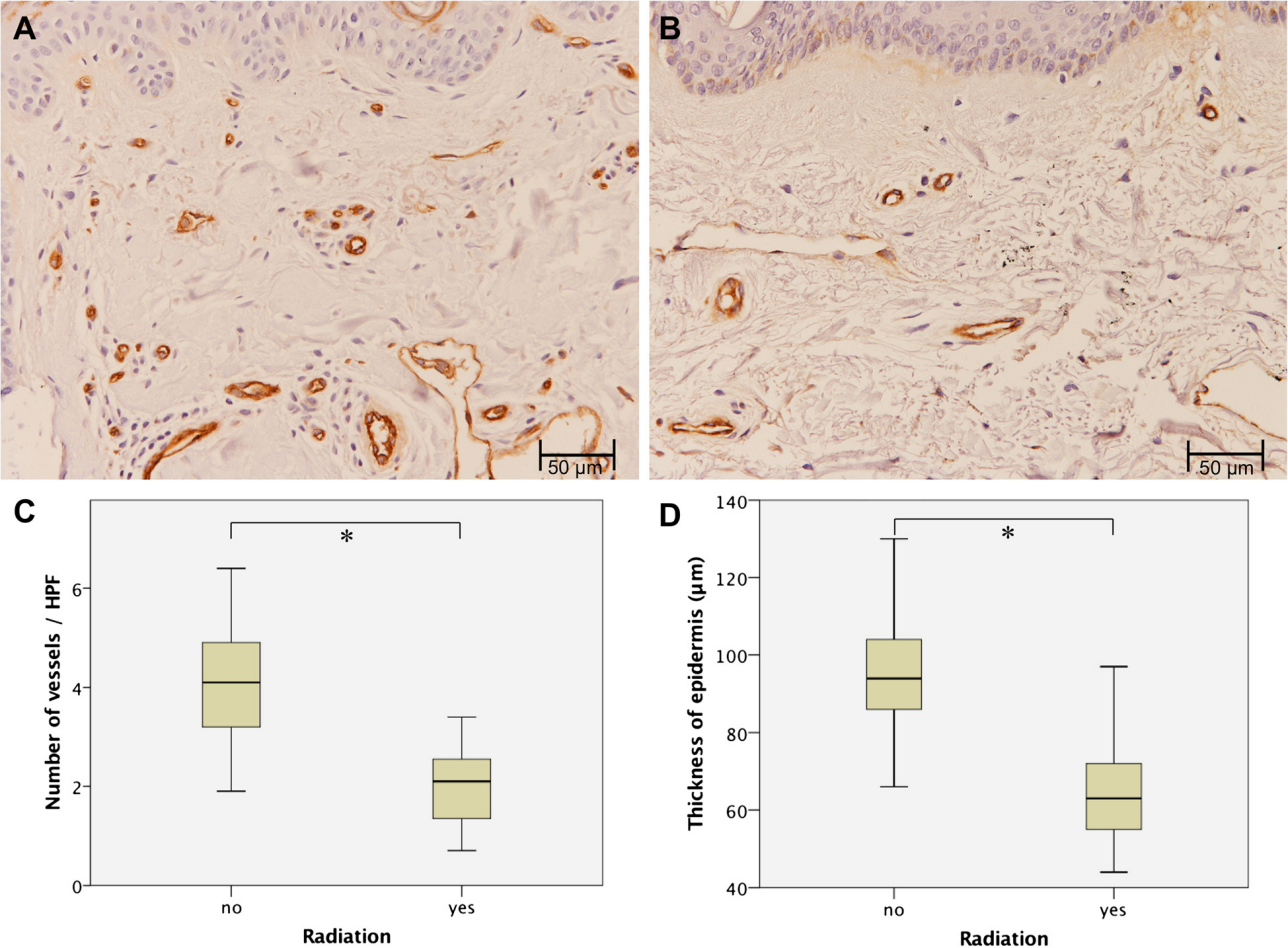
Results of immunohistochemical analysis. **a** Histological photograph of vWF staining in non-irradiated cervical skin. Scale bar = 50 μm. **b** Histological photograph of vWF staining in irradiated cervical skin. Scale bar = 50 μm. (**c** + **d**) Boxplot diagrams visualizing the number of stained vessels per high power field (HPF) (**c**); and the measured thickness of the epidermis (**d**). Given is the median value, (with the 75th percentile and the 25th percentile) calculated over ten HPFs in patients with (*n =* 27) and without RT (*n =* 57). The range is shown as a vertical line; extreme values are excluded. *P*-values were calculated by means of the Mann–Whitney test. Statistical significance between groups could be shown (both *: *p* = 0.000)

In HE-stained sections, the thickness of the epidermis was measured (Fig. 2a-c). Immunohistochemical staining for vWF was analyzed according to the amount of vessels per HPF as well as the size of the vessels. For all investigated approaches, 10 HPFs were analyzed by two independent investigators, and a mean value was calculated. Non-irradiated skin showed a regular thickness of the epidermis, whereas preoperatively irradiated tissue showed histological signs of epidermal atrophy with statistically significant differences in the thickness of the epidermis (98.6 ± 20.3 μm vs. 63.8 ± 12.8 μm; *p* = 0.000; Fig. 3d). With regard to vessel density in immunohistochemical staining for vWF, a decrease in irradiated tissue versus skin that has not been exposed to RT could be observed (2.0 ± 0.7 vs. 4.4 ± 1.6; *p* = 0.000; Fig. 3c). Analysis of vessels size did not reveal statistically significant differences between the study groups. Photomicrographs of immunostaining for vWF in tissue specimens with and without a history of previous RT are shown in Fig. 3a and b.

### RT-PCR

**Fig. 4:**
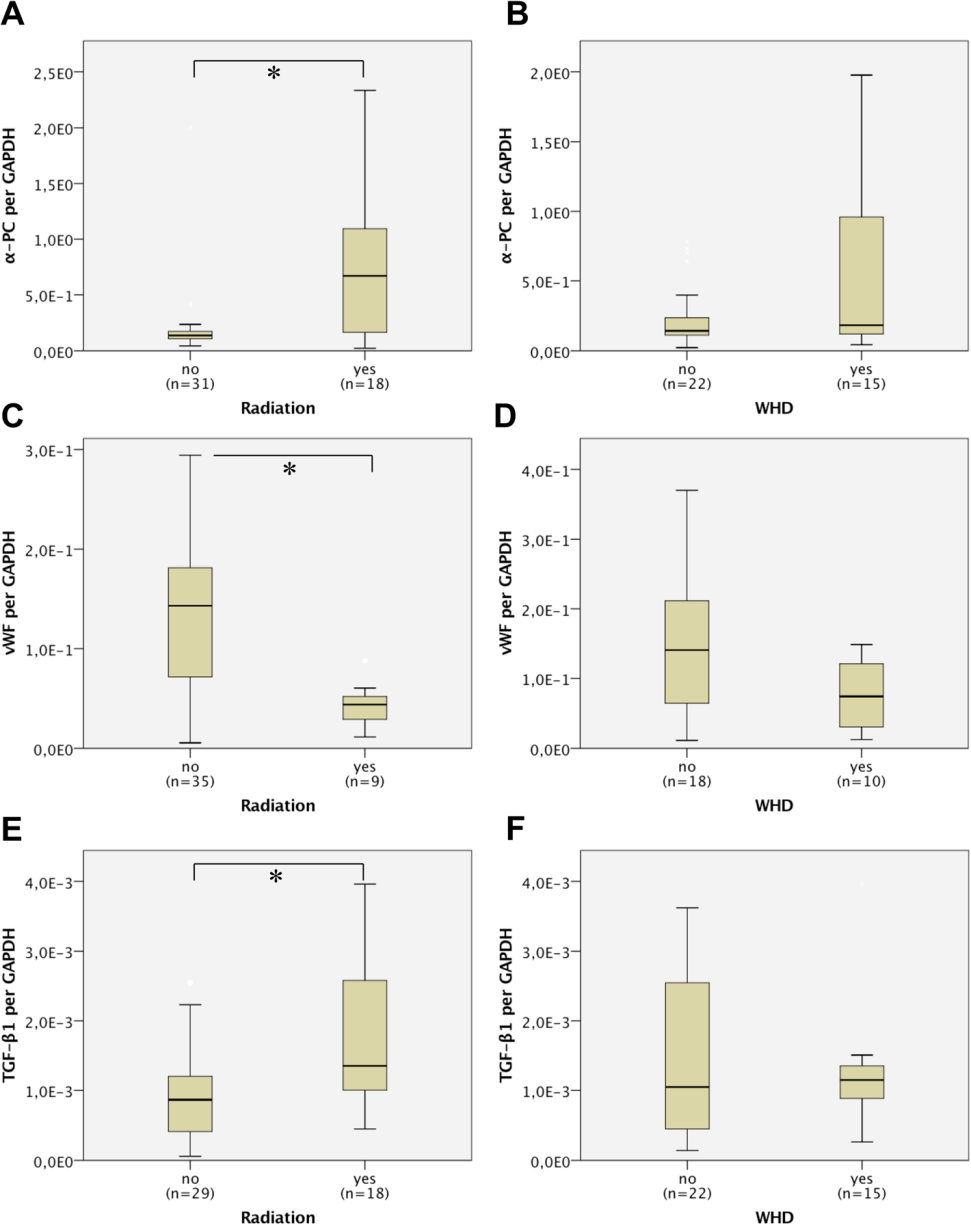
Boxplot diagrams visualizing the gene expression data of the investigated genes α-Procollagen: **a**: patients with (*n =* 18) and without (*n =* 31) a history of Radiation Therapy (RT); **b**: patients with (*n =* 15) and without (*n =* 22) occurrence of postoperative Wound Healing Disorders (WHD). VWF: **c**: patients with (*n =* 9) and without (*n =* 35) a history of RT; **d**: patients with (*n =* 10) and without (*n =* 18) occurrence of WHD. TGF-β1: **e**: patients with (*n =* 18) and without (*n =* 29) a history of RT; **f**: patients with (*n =* 15) and without (*n =* 22) occurrence of WHD. Measured by real-time RT-PCR in cervical tissue samples. Given is the median value, (with the 75th percentile and the 25th percentile). The range is shown as a vertical line; extreme values are excluded. *P*-values were calculated by mean of the Mann–Whitney test. Statistical significance between groups could be shown (all *: *p* < 0.005). WHD = wound healing disorder

The expression of all the investigated genes was analyzed according to a history of RT and the occurrence of postoperative WHDs. α-PC showed a statistically significant higher expression in previously (more than 91 days) irradiated tissue than in the non-irradiated controls (9.1E-01 ± 1.1E-01 vs. 2.1 ± 3.4E-01; *p* = 0.002; Fig. 4a). However, the expression of α-PC in skin samples that had developed postoperative WHD was increased, but without statistical significance (7.4E-01 ± 1.2E-01 vs. 2.5E-01 ± 2.4E-01; *p* = 0.067; Fig. 4b). Results of RT-PCR are displayed in Table 3.

**Table 3:** Real-time RT-PCR results in groups with/without preoperative RT and with/without postoperative cervical WHDs

Gen	Median expression per GAPDH			Median expression per GAPDH		
	RT	No RT		WHD	No WHD	
	N (%)	N (%)	*p* Value	N (%)	N (%)	*p* Value
α-Procollagen	9.1 E-01	2.1 E-01	0.002*	7.4 E-01	2.5 E-01	0.067
	18 (36.7)	31 (63.3)		15 (40.5)	22 (59.5)	
TGF-β1	2.3 E-03	1.1 E-03	0.012*	1.2 E-03	2.1 E-03	0.245
	18 (38.3)	29 (61.7)		15 (40.5)	22 (59.5)	
vWF	4.3 E-02	1.5 E-01	0.005*	7.8 E-02	1.6 E-01	0.080
	9 (20.5)	35 (79.5)		10 (35.7)	18 (64.3)	

Abbreviations: *WHD* wound healing disorder, *RT* radiation therapy, *RT-PCR* reverse-transcription polymerase chain reaction

Data in parentheses are percentages, unless noted otherwise; *statistically significant difference at α=0.0

Similar results were observed for the expression analysis of TGF-β1, with a statistically significant difference between the irradiated and non-irradiated group (2.3E-03 ± 2.7E-03 vs. 1.1E-03 ± 1.2E-03; *p* = 0.012; Fig. 4c). An increased expression within the group without clinical signs of WHDs during follow-up could be seen, but this showed no significant difference (2.1E-03 ± 2.7E-03 vs. 1.2E-03 ± 8.3E-04; *p* = 0.245, Fig. 4d). Expression analysis for vWF in preoperatively irradiated tissue exhibited a statistically significant increase in the RT group (1.5E-01 ± 1.1E-01 vs. 4.3E-02 ± 2.2E-02; *p* = 0.005; Fig. 4e). An increased expression in tissue with a clinical WHD in follow-up could also be observed in comparison with the non-irradiated control group (7.8E-02 ± 5.1E-02 vs. 1.6E-01 ± 1.3E-01; *p* = 0.080; Fig. 4f).

## Discussion

The use of RT in the interdisciplinary treatment of HNC is accompanied by acute side effects, such as dermatitis, nausea, edema, mucositis, and swallowing disorders, and long-term side effects including xerostomia, osteoradionecrosis, and even the risk of secondary radiation-induced malignancies [27]. The individual characteristic of RT-induced side effects is influenced by the total irradiation dose, overall treatment time, and tumor localization [28]. Furthermore, several years ago, fibrosis was demonstrated to be a significant side effect that could impair neck movement. Radiation-induced fibrosis (RIF) and consequently dysfunction of microcirculation are also structural effects of RT [29]. This is most easily assessed in skin but is also true for other organs within the radiation field, as RIF can result in inadequate ventilation, muscle stiffness, decreased cardiac output, and delayed wound healing [30, 31]. Previous studies have shown the essential role of inflammatory cytokines such as TGF-β1 in the pathogenesis of RIF, as fibrosis is known to be caused by excess collagen and alterations in the extracellular matrix (ECM) [31]. TGF-β1 is reported to promote fibrosis and to suppress vascularization during wound healing and is considered to play a key role in the molecular pathogenesis of RIF [31].

Wound healing in previously irradiated skin is defective and can lead to prolonged morbidity because of wound dehiscence, infections, failure of reconstructive skin or free flaps, skin necrosis, and persistent fistula formations. Histopathological characteristics of impaired wound healing after RT include RIF and impaired neovascularization [32]. Clinical parameters such as oxygen saturation, relative hemoglobin concentration, and blood velocity do not show significant differences between patients with or without WHD following RT, as our own preliminary studies suggest [15]. Nevertheless, only a few reports in the literature involve the investigation of the effects of RT in terms of WHD in HNC patients.

This current study investigates the role of marker proteins such as TGF-β1, vWF, and α-PC in angiogenesis and fibrosis in cervical tissue samples in patients with and without preoperative RT. Moreover, the identification of key factors in the complex pathophysiology of debilitating wound healing after RT might represent the foundation for the evaluation of a potential intersection for therapeutic interventions in the future. The identification of TGF-β1 as an essential cytokine in RIF, α-PC as a marker of structural changes in the microarchitecture of the skin, and vWF as a significant tool in the characterization of vascularization mechanisms, i.e., as specific markers that help to understand the effects of RT, allows them to be used for immunohistochemical and molecular analysis. Analysis of clinical data obtained preoperatively and during follow-up shows a significantly increased relative risk of patients developing a WHD after RT. Length of in-hospital stay also shows significant differences between irradiated and non-irradiated patients. WHDs occurred earlier in patients with a positive history of exposure to ionizing radiation. In brief, these results underline the importance of an increased clinical sensitivity in irradiated patients who have to undergo surgery within the former radiation field. Special caution should be paid to sufficient wound closure, including the careful bipolar sealing of small vessels, the application of multi-layered closure (platysma, subcutaneous, and superficial skin suture), and the use of perioperative anti-microbial prophylaxis. This seems especially important as a reduced rate of postoperative WHDs after neck dissection not only cuts down individual morbidity, but also reduces the time of in-hospital stay and effort during wound closure and instantly counters any additional expenses. These clinical findings and the possibility of identifying patients at risk preoperatively by easily applicable, accessible, patient compliant and non-invasive tools, such as tissue spectrometry and laser Doppler flowmetry, reveals the importance of sufficient surgical care in patients after RT of the head and neck [15]. Moreover, the fact that WHDs occurred earlier in preoperatively irradiated patients especially emphasizes the first three to six postoperative days. This calls for a close clinical evaluation and the low-threshold use of postoperative antibiotics, particularly in irradiated patients.

Tissue specimens were collected, and immunohistological staining against vWF was performed. The results of such immunostaining against vWF and the detection of vessels in the collected skin specimens demonstrate significant differences between patients with and without RT. Moreover, significant differences in skin fibrosis and epidermal thickness have been histologically established in the two study groups. These results bolster reports on skin atrophy, fibrosis, and a reduced neovascularization in the context of WHD following RT as described in the literature [32].

Expression analysis of markers in fibrosis and angiogenesis were carried out subsequently in order to gain insight into the molecular pathways that account for the structural changes in irradiated skin and that eventually lead to WHDs. A statistically significant increase in the expression of α-PC per GAPDH can be found in study groups with a history of RT. This supports findings that radiation-induced dermal fibrosis is associated with an increased expression of α-PC [33, 34].

Collagen, synthesized by fibroblasts shows an increased expression in the context of inflammatory reactions, as others have demonstrated [35]. These findings are congruent with the results of the increased expression analysis of α-PC in patients with a WHD during follow-up. Whereas fibroblasts in hypertrophic scars do not show an increased expression of α-PC in experiments in vitro, an significant increase in scleroderma lesions of Type I collagen could be found [36].

The results of vWF in RT-PCR expression analysis have revealed a significant increase in control tissue samples compared with those after RT and an increase within the control group in tissue samples that developed WHD during follow-up. However, as yet, no statistically significant differences have been detected. These results reflect the enormous influence of neovascularization processes in irradiated skin tissue. A sufficient supply of epidermal tissue with oxygen in terms of adequate vascularization is especially important in skin that has undergone radiation-induced fibrosis, in order to prevent the incidence of WHD following surgery to the neck during follow-up. However, not only WHDs but also other numerous histopathological changes with regard to the late side effects of RT are affected by an impaired neovascularization [32].

Expression analysis of TGF-β1 has revealed significant differences between previously irradiated patients and those without a history of RT. The effect of TGF-β1 in RIF has been subject to other studies in the literature [31, 37]. An increase of TGF-β1 in patients with a WHD during follow-up can be observed, but no significant difference has been measured in statistical analysis. These findings are compatible with those from other study groups. Schultze-Mosgau et al. have demonstrated the relationship between TGF-β1 and the occurrence of RIF in a murine animal model and *in vitro*. The inhibition of TGF-β1 activity has a substantial effect on collagen synthesis and on matrix-degrading enyzmes such as matrix metalloproteinase-1 and matrix metalloproteinase-3 [37]. The application of anti-TGF-β1 antibodies results in improved healing of free tissue flaps in previously irradiated tissue and a reduced expression of pro-fibrotic proteins such as α-PC [37].

Our current study involves an investigation of the influence of radiation-induced skin fibrosis on WHDs in a prospective study setting. Tissue specimens have been obtained during surgical approaches to the cervical lymph nodes and analyzed by using immunohistochemistry and RT-PCR. The occurrence of WHDs has been evaluated clinically during a postoperative follow-up of 30 days. The increased expression of marker proteins such as TGF-β1 supports pathogenetic theories on the development of RIF. However, the molecular mechanism of skin fibrosis in the close relationship to the occurrence of WHDs during clinical follow-up are seldomly studied. The current study adds important information about the expression profiles of marker proteins of RIF. We have found an increase of TGF-β1 and α-PC expression in irradiated skin and in tissue samples with an onset of WHD immediately after surgery. VWF shows consequently a decrease in irratiated tissues and occurrence of WHDs.

## Conclusions

The results of this current study emphasize the importance of cytokines in skin fibrosis and create perspectives of the possible clinical applications of specific antibodies. This should be investigated in further studies.

However, the need for accurate and thoroughly surgery in patients after RT, especially in head and neck procedures, is highlighted as the clinical data suggest. This could act as an attentive reminder to those involved in the complex surgical care of this specific group of patients in the need for meticulous oncologic rehabilitation.
